# MurSS: A Multi-Resolution Selective Segmentation Model for Breast Cancer

**DOI:** 10.3390/bioengineering11050463

**Published:** 2024-05-07

**Authors:** Joonho Lee, Geongyu Lee, Tae-Yeong Kwak, Sun Woo Kim, Min-Sun Jin, Chungyeul Kim, Hyeyoon Chang

**Affiliations:** 1 Deep Bio Inc., Seoul 08380, Republic of Korea; joonho.lee@deepbio.co.kr (J.L.); gglee@deepbio.co.kr (G.L.); tykwak@deepbio.co.kr (T.-Y.K.); swkim@deepbio.co.kr (S.W.K.); 2 Department of Pathology, Bucheon St. Mary’s Hospital, College of Medicine, The Catholic University of Korea, Seoul 14647, Republic of Korea; seasy@hanmail.net; 3 Department of Pathology, Korea University Guro Hospital, Seoul 08308, Republic of Korea; idea1@hanmail.net

**Keywords:** multi-resolution, segmentation, selective segmentation method, breast cancer

## Abstract

Accurately segmenting cancer lesions is essential for effective personalized treatment and enhanced patient outcomes. We propose a multi-resolution selective segmentation (MurSS) model to accurately segment breast cancer lesions from hematoxylin and eosin (H&E) stained whole-slide images (WSIs). We used The Cancer Genome Atlas breast invasive carcinoma (BRCA) public dataset for training and validation. We used the Korea University Medical Center, Guro Hospital, BRCA dataset for the final test evaluation. MurSS utilizes both low- and high-resolution patches to leverage multi-resolution features using adaptive instance normalization. This enhances segmentation performance while employing a selective segmentation method to automatically reject ambiguous tissue regions, ensuring stable training. MurSS rejects 5% of WSI regions and achieves a pixel-level accuracy of 96.88% (95% confidence interval (CI): 95.97–97.62%) and mean Intersection over Union of 0.7283 (95% CI: 0.6865–0.7640). In our study, MurSS exhibits superior performance over other deep learning models, showcasing its ability to reject ambiguous areas identified by expert annotations while using multi-resolution inputs.

## 1. Introduction

Breast cancer is a prevalent cancer among women worldwide [1]. In the United States, breast cancer is the second leading cause of cancer-related deaths in women [2]. Breast cancer is diagnosed by analyzing pathological features such as tumor growth patterns and cytologic characteristics. The most common type is invasive ductal carcinoma (IDC). IDC accounts for approximately 70–80% of all breast cancers [3]. Accurately measuring invasive tumor size is essential in breast cancer prognosis. However, in cancer lesions, IDC may coexist with ductal carcinoma in situ (DCIS), sometimes showing intermingled patterns. Therefore, measuring invasive tumor size via microscopic examination may be challenging. The presence of an extensive intraductal component is a critical factor in predicting breast cancer prognosis. The rapid growth of digital pathology has opened up possibilities for AI to aid pathologists in diagnosis. Tumor area estimation techniques can assist pathologists by providing more accurate measurements of invasive tumors [4,5,6].

Due to their gigapixel size and high resolution, whole-slide images (WSIs) require high computational costs. For example, WSIs from The Cancer Genome Atlas (TCGA) dataset [7], which are widely used in pathological image analysis, have a resolution that is a gigapixel in size, significantly higher than that of standard image datasets. As a result, each WSI is divided into smaller pieces, which are then used to train deep learning models. In this case, we need to consider the receptive field, which is the area of the input image that influences the output [8]. When extracting a patch at a higher resolution, the contextual information from the wider area is not utilized as the tissue around the patch is not included in the receptive field. This fact limits the use of high-resolution content information and omits low-resolution contextual information.

In computer vision, previous deep learning models have primarily been designed for high-resolution, singular images, as these images are typically compact and do not require tiling into smaller patches. The authors of [9] proposed U-Net to accurately segment cellular structures from medical image backgrounds. U-Net uses low-level features during the high-to-low downsampling process and combines them into same-resolution high-level features during the low-to-high upsampling process through skip connections. Additionally, U-Net employs single-resolution images as its input. The authors of [10] proposed DeepLabV3, which utilizes dilated convolution to expand the receptive field, thereby leveraging the broader contextual information. Most segmentation tasks compress high-resolution images by passing them through CNN layers and then employing an upsampling method. However, the authors of [11] proposed HRNet, a high-resolution network that maintains high resolutions throughout the entire process and progressively adds feature maps of lower resolutions in parallel as the layers deepen; this technique is described as multi-resolution streams in parallel. As a result, HRNet can accurately segment regions, even in complex images, since both high- and low-resolution features are combined. Various models are being developed that utilize connections between features of different scales and expand the receptive field, aiming to work with multi-scale feature maps or high-resolution images. Nowadays, research focuses on combining feature information from images of various resolutions. The authors of [12] argued that more detailed areas can be observed as the resolution increases, but overall morphological features, such as the background, are missed. The authors of [13] proposed an image cascade network (ICNet) using cascade feature fusion. ICNet is trained by designing networks for three different-resolution images and combining them. In this way, the critical feature information from each resolution is used. The authors of [14] proposed a deep multi-magnification network (DMMN) for multi-class breast cancer image segmentation consisting of three U-Nets. They placed three images of different resolutions in the encoder of U-Net, concatenating the features. Various studies have been conducted to analyze and combine images of different resolutions [15,16].

Furthermore, owing to the morphological similarities between DCIS and IDC, the annotations of pathologists can differ depending on their proficiency. This could lead to the deep learning model producing uncertain predictions. Therefore, handling these uncertainties is essential for obtaining better diagnostic models. To solve this problem, researchers have been working on statistical uncertainty for a long time. For example, the reject option has been applied to support vector machine, nearest neighbor, and k-nearest neighbor models [17,18,19]. When considering selective prediction in neural networks, the most common method is to find a rejection threshold from a pre-trained model with all the data and to train the model using that threshold [20,21]. This approach involves training using the entire dataset and determining a rejection threshold using outputs that yield ambiguous results. Subsequently, by using this rejection threshold, data are excluded during training. A drawback of this method is that it requires two rounds of training. Therefore, recent studies have devised methods to simultaneously determine the rejection threshold and exclude data based on that threshold during training [22]. In addition, methods using Monte Carlo dropout to reject uncertain data [23] and methods using softmax response are available.

Based on these studies, we have developed a model for segmenting DCIS and IDC in breast tissue. We introduce the multi-resolution selective segmentation (MurSS) model, which uses low- and high-resolution patches to leverage multi-resolution features and reject ambiguous regions using the selective segmentation method (SSM). The major contributions of this study are summarized as follows:

- We incorporate a large field of view as an explicit context within our model’s architecture, improving the cancer lesion segmentation performance.
- We address the challenge of ambiguity in segmentation tasks for pathological images through the strategic implementation of selective segmentation methods.

## 2. Materials and Methods

### 2.1. Materials

For MurSS’s training and evaluation, we used 1276 hematoxylin and eosin (H&E) stained WSIs. These datasets consist of 1181 TCGA [7] breast invasive carcinoma (TCGA-BRCA) and 95 Korea University Medical Center, Guro Hospital (KUMC-Guro), BRCA H&E stained WSIs. Three pathologists working at different institutions annotated the WSIs for benign, DCIS, and IDC. We created training, validation, and test datasets using majority voting for three different sets of annotated data. From this, 87 slides were excluded from the dataset due to slide quality issues that could affect the model’s performance [24]. As a training dataset, 876 TCGA-BRCA WSIs were randomly selected and used, while the remaining 218 TCGA-BRCA WSIs were used for validation. The test performance was evaluated using 95 WSIs from KUMC-Guro, utilizing the model with the highest performance on the validation set. KUMC-Guro BRCA H&E stained WSIs were collected from early breast cancer patients with pN0, hormone receptor-positive, and human epidermal growth factor receptor type 2 (HER2)-negative statuses. The data used in this study were collected from January 2014 to July 2019 and scanned using an Aperio AT-2 scanner. If the patient had already undergone chemotherapy before surgery or the pathologist determined that the H&E slide condition was unsuitable for clinical histological and pathological trials, the relevant data were not collected.

We divided each slide into 544 × 544-pixel patches for training, validation, and testing. The images were of high, mid, and low resolutions with magnifications of 5.0×, 2.5×, and 1.25×, respectively. We extracted patches at a 5.0× magnification and then used the central coordinate values to extract patches at magnification levels of 2.5× and 1.25×. Consequently, in the case of the 2.5× and 1.25× magnification patches, overlapping areas existed. Using all patches from different resolutions, we randomly cropped them to a size of 512 × 512 pixels. To overcome the data imbalance problem, we oversampled the DCIS patches nine times. The same patches were utilized multiple times based on the oversampling ratio. Subsequently, hard augmentations were implemented, including flipping, rotating, blurring, cropping, color jittering, and so forth. Table S3 presents the experimental results obtained with varying oversampling ratios.

### 2.2. Methods

This section describes the key elements of the multi-resolution selective segmentation (MurSS) model. MurSS consists of the multi-resolution adaptive normalization (MurAN) model, which receives high-resolution and low-resolution images as input and produces pixel-level classification output, and the selective segmentation method (SSM), which selects the pixel-level uncertainty-based classification output. MurAN is introduced in Section 2.2.1, and the SSM is introduced in Section 2.2.2. Finally, in Section 2.2.3 we briefly explain our evaluation metrics.

#### 2.2.1. Multi-Resolution Adaptive Normalization

**Figure 1:**
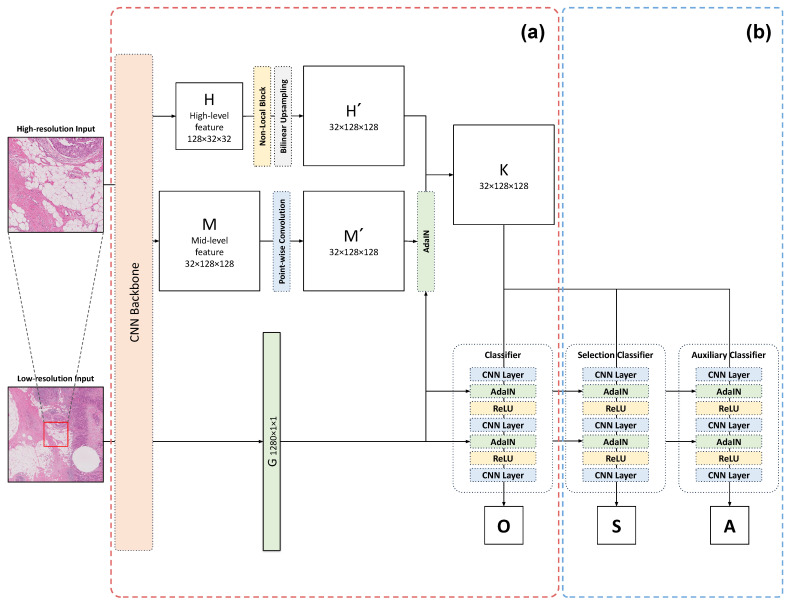
The multi-resolution selective segmentation (MurSS) model consists of 2 parts. (**a**) The multi-resolution adaptive normalization (MurAN) model extracts high-level (*H*) and mid-level (*M*) features from high-resolution patches. *G* represents the extracted contextual information from low-resolution patches. *G* is combined with feature maps (*K*) through the adaptive instance normalization (AdaIN) blocks. Finally, the classifier creates the pixel-level classification output (*O*). (**b**) The selective segmentation method (SSM) is added to MurAN. MurSS uses three classifiers, while MuRAN uses only one classifier that produces the original output (*O*). *O* is the original output that is produced by the prediction function, *f*. The selection output (*S*) is produced by the selection function, *g*, to determine the selection coverage. The auxiliary function, *h*, is only used for training to generalize the loss and produce an auxiliary output (*A*).

The architecture of our proposed model is shown in Figure 1. It processes two inputs of different resolutions, utilizing a shared-weight CNN model, termed the ’backbone’, to extract multi-resolution features. The model generates mid- and high-level features through the backbone when processing high-resolution input. Conversely, for low-resolution input, after passing through the backbone, the model obtains contextual information regarding the overall style of the image.

High-level, high-resolution features pass through the non-local block layer to incorporate patch-wise global features. The non-local block, also known as the self-attention mechanism, is a method proposed by [25]. Non-local blocks can capture different types of spatial information, leading to better performance [26,27,28]. Using the non-local block and bilinear upsampling, we obtain $H^{\prime }\in R^{B\times C\times H\times W}$, with a size of B×32×128×128. *B*, *C*, *H*, and *W* denote the batch size, channel, height, and width of the feature. Mid-level features, *M*, have a shape of 32×128×128, and these features go through a pointwise convolution layer and return $M^{\prime }\in R^{C\times H\times W}$ with the same shape as *M*. Then, $M^{\prime }$ is combined with the contextual information $G\in R^{C\times H\times W}$ with a size of 128×1×1. If we concatenate $M^{\prime }$ and *G*, the representation of each pixel may be different due to the different sizes of the features. This may cause performance degradation in the segmentation task. To solve this problem, we used adaptive instance normalization (AdaIN), as proposed in [29]. AdaIN extends direct application in style transfer by normalizing the content of an image’s features and then applying the style image’s mean and standard deviation. This approach allows AdaIN to effectively transfer the style characteristics to the content image, maintaining its structure while adopting the style attributes. AdaIN scales $M^{\prime }$’s normalized content input with *G* and shifts it with *G* without any learnable affine parameters. This computation is based on batches, meaning it is carried out independently for each image within a batch. In short, AdaIN performs style transfer in the feature space by transferring feature statistics, specifically the channel-wise mean and standard deviation. Through AdaIN, we obtain $M^{\prime \prime }$ of size 32×128×128, represented as $M^{\prime \prime }\in R^{C\times H\times W}$. Finally, the patch-wise global feature from the non-local block, $H^{\prime }$, and $M^{\prime }$ are merged through a concatenation process. Afterward, we pass it through a classifier and produce the final output.

#### 2.2.2. Selective Segmentation Method

To overcome the data uncertainties derived from the morphological similarities of DCIS and IDC, as well as the possibility of differing annotations by pathologists, we used the selective segmentation method (SSM). By using the SSM, we can automatically reject ambiguous regions. SelectiveNet [22] enables the model to reject inputs that frequently yield erroneous results, thereby mitigating the potential for uncertain predictions during both the inference and training processes. Inspired by SelectiveNet, we developed the SSM for any segmentation task. The SSM produces the output of MurAN and two additional outputs: the selection and the auxiliary output. The authors of [22] stated that the performance of a selective method can be quantified using both the coverage ratio and risk. We defined the empirical coverage risk as

(1) $$r\left(I_{high},I_{low},y\right)=\frac{\sum_{i=1}^{m}\sum_{j=1}^{n}l\left(f{\left(I_{high},I_{low}\right)}_{ij},y_{ij}\right)g{\left(I_{high},I_{low}\right)}_{ij}}{\sum_{i=1}^{m}\sum_{j=1}^{n}g{\left(I_{high},I_{low}\right)}_{ij}}$$

where $I_{high}$ and $I_{low}$ are the high- and low-resolution patches; *y* is the ground-truth label; l(a,b) is the pixel-wise cross-entropy loss; *a* is the output of the model; and *b* represents the images annotated by the pathologists. Also, *f* is the prediction function that produces the output and *g* is the selection function that produces the selection output. Equation (1) shows the empirical coverage risk regarding the selective region based on the coverage ratio. Also, the quadratic penalty term, $L_{Penalty}=max{\left(0,T-\frac{1}{mn}\sum_{i=1}^{m}\sum_{j=1}^{n}g{\left(I_{high},I_{low}\right)}_{ij}\right)}^{2}$, is added, as shown in Equation (2).

(2) $$L_{Select}=r\left(I_{high},I_{low},y\right)+\lambda L_{Penalty}$$

where *T* is the pre-defined value from 0 to 1 that indicates the coverage ratio the model aims to achieve and λ controls the relative importance of the constraints. We train MurSS to reduce the empirical selective risk to minimize $L_{Select}$. To determine the optimal empirical coverage risk, certain pixels must be rejected under the condition that the percentage of non-rejected pixels meets or exceeds the coverage ratio *T*. When $\frac{1}{mn}\sum_{i=1}^{m}\sum_{j=1}^{n}g{\left(I_{high},I_{low}\right)}_{ij}<T$, the quadratic penalty term will be greater than 0, reducing $L_{Select}$ and increasing the size of rejection regions. Therefore, there will be more opportunities to reject uncertain areas. Otherwise, when $\frac{1}{mn}\sum_{i=1}^{m}\sum_{j=1}^{n}g{\left(I_{high},I_{low}\right)}_{ij}\geq T$, the quadratic penalty term will be 0, allowing the model to focus more on reducing the risk within the coverage area without reducing the size of rejection regions. However, if we calculate the loss based only on the coverage region, the model may overfit to the selected data. To address this, we use $h(I_{high},I_{low})$, which represents the auxiliary prediction, to compute the auxiliary prediction risk during the training process. Therefore, our model’s loss is defined as

(3) $$L_{Total}=\alpha L_{Select}+\left(1-\alpha \right)L_{Aux}$$

where $L_{Aux}=\frac{1}{mn}\sum_{i=1}^{m}\sum_{j=1}^{n}l\left(h{\left(I_{high},I_{low}\right)}_{ij},y_{ij}\right)$. From this, α is the hyperparameter used to control the ratio of empirical risk and auxiliary prediction risk. Also, in $L_{Aux}$, l(a,b) is the pixel-wise cross-entropy loss, where *a* is the auxiliary output of the model and *b* represents the images annotated by the pathologists.

#### 2.2.3. Evaluation Metrics

To evaluate the performance of our proposed model, MurSS, against other segmentation models, we use two metrics: pixel-level accuracy (accuracy) and Intersection over Union (IoU). Accuracy measures the percentage of correctly classified pixels in the image (Equation (4)). The IoU is calculated as the area of the intersection over the union of the ground truth ($S_{y}$) and prediction area ($S_{O}$). The formulas for accuracy and the IoU are shown in Equation (5).

(4) $$Accuracy=\frac{TP+TN}{TP+TN+FP+FN}$$

(5) $$IoU\left(S_{O},S_{y}\right)=\left|\frac{S_{O}\cap S_{y}}{S_{O}\cup S_{y}}\right|=\frac{TP}{TP+FP+FN}$$

Regarding the metrics for IDC, the following confusion matrix (Table 1) shows examples of TPs, FNs, FPs, and TNs. The confusion matrix is different from binary cases.

The 95% confidence intervals (CI) for accuracy and mIoU were measured using the bootstrap method. A bootstrap sample was generated via random sampling with replacement from the original test dataset, ensuring each bootstrap sample was the same size as the original test dataset. This process was independently and randomly repeated 1000 times. For each bootstrap sample, accuracy and mIoU were calculated. The bootstrap 95% confidence intervals for each of the two metrics were then estimated from these repeated measurements.

## 3. Results

This section provides a concise overview of the research, presenting the conclusions drawn from the evidence analyzed. The checkpoint obtained from the most optimal epoch of the validation dataset was used for all evaluations. The inference results on the test dataset were then measured based on these checkpoints. The performance of the validation set is shown in Table S1.

Table 2 shows a quantitative analysis of various segmentation models, including the proposed MurAN and MurSS, with predefined coverage ratios of 0.95, 0.90, and 0.80, against established models such as U-Net, HRNet, DeepLabV3, ICNet, and DMMN. The performance metrics include pixel-level accuracy (accuracy), mean Intersection over Union (mIoU), and class-specific IoUs for benign, DCIS, and IDC. To evaluate the performance of the single-resolution model, we used U-Net and compared the results obtained from high, mid, and low resolutions. The high-resolution U-Net model exhibited higher mIoU values than the other U-Net models, with increases ranging from 0.0154 to 0.0236. Based on these results, we evaluated the performance of HRNet and DeepLabV3 using the high-resolution patch with the best performance and compared it with the other multi-resolution models, MurAN and MurSS.

**Figure 2:**
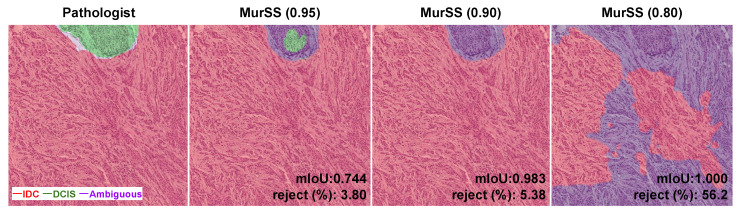
Visualization results of MurSS. Comparison between pathologists and MurSS with different coverage ratios. Green areas represent DCIS, red areas represent IDC, and purple areas represent the rejected regions. MurSS with a coverage ratio of 0.80 shows the highest mIoU, but it rejects 56.2% of regions. The visualization results show it is unreliable for diagnosis.

The performance of MurSS improved, indicating its ability to automatically classify uncertain areas that may be difficult or ambiguous for pathologists. The best numerical performance was achieved with a coverage ratio of 0.80 (${\mathrm{MurSS}}_{0.80}$). However, this may not provide a reliable diagnosis, as the measurement was taken with a coverage ratio of 0.80, indicating that 20% of regions were ambiguous. In the visualization presented in Figure 2, the purple regions indicate that ${\mathrm{MurSS}}_{0.80}$ rejected too many regions. ${\mathrm{MurSS}}_{0.80}$ rejected a significant amount of DCIS and IDC. The trained MurSS with a coverage ratio of 0.95 (${\mathrm{MurSS}}_{0.95}$) accurately identified the obvious areas while only rejecting some of the ambiguous regions. Therefore, we used ${\mathrm{MurSS}}_{0.95}$ for our qualitative analysis.

The proposed ${\mathrm{MurSS}}_{0.95}$ achieved the highest performance compared to other models by rejecting 5% of WSI regions, resulting in an mIoU of 0.7283 and an accuracy of 96.88%. Additionally, the proposed MurAN model achieved an mIoU of 0.7055 and an accuracy of 95.88%, which was the second-highest overall measure after ${\mathrm{MurSS}}_{0.95}$. When examining the class-specific IoU, ${\mathrm{MurSS}}_{0.95}$ and MurAN showed the best performance for benign and IDC, while HRNet and DeepLabV3 showed the best performance for DCIS. Also, a statistical analysis was conducted to verify ${\mathrm{MurSS}}_{0.95}$’s superiority. The results of the analysis confirm the superiority of ${\mathrm{MurSS}}_{0.95}$ and MurAN. Detailed results of our statistical analysis are presented in Table S2.

**Figure 3:**
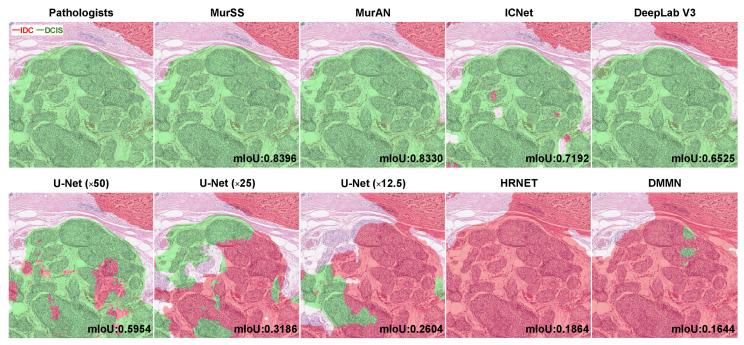
Visualization results. Comparison between pathologists and deep learning models. The mIoU was measured at the patch level. Green areas represent DCIS, and red areas represent IDC.

Figure 3 shows the visualization result and mIoU of each patch with multiple deep learning models. The green areas indicate the regions where the models predicted DCIS, and the red areas indicate the regions where models predicted IDC. Generally, ${\mathrm{MurSS}}_{0.95}$, MurAN, DeepLabV3, and HRNet performed well regarding mIoU. However, MurAN, DeepLabV3, and HRNet exhibited overconfidence in their results, leading to misjudgments of IDC and DCIS. Further visualization results are presented in Figure S1a–c.

## 4. Discussion

This study proposes a segmentation model for accurate breast cancer diagnosis. MurSS attempts to overcome the limitations of existing methods by combining contextual and content information from various resolutions using adaptive instance normalization. Additionally, it effectively performs segmentation for uncertain areas using the SSM. To measure the uncertainty, the Intersection over Union (IoU) from DCIS annotations by two pathologists was calculated. The slide-level IoU of DCIS was 0.44 between the two pathologists, showing that DCIS can be interpreted differently.

### 4.1. Visualization Explanation

**Figure 4:**
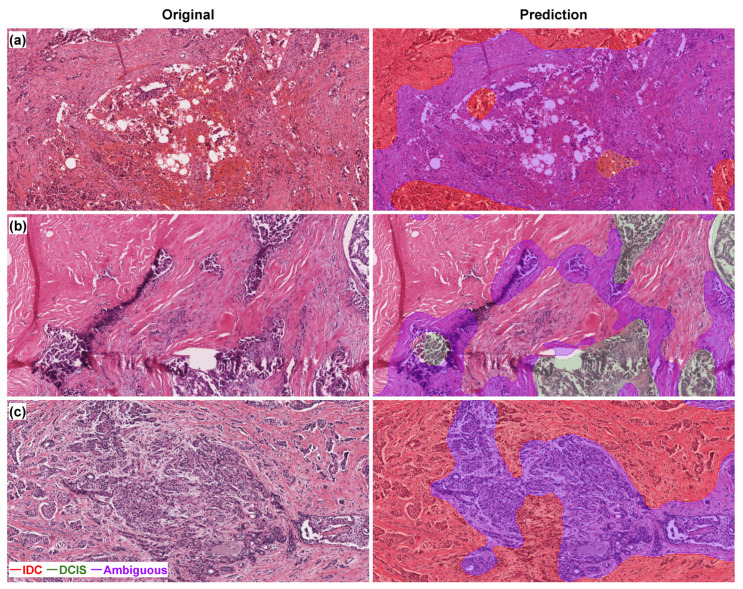
Visualization results of MurSS. The model identified regions with tissue artifacts as ambiguous (**a**). The ambiguous region also showed tumor cell nests without a definite myoepithelial cell layer (**b**). The model also identified lesions in the area where sclerosing adenosis and small tumor cell nests were intermingled as vague (**c**). Slide regions were classified as benign (no color), IDC (red), DCIS (green), and ambiguous (purple).

The visualization results are provided in Figure 4. MurSS identified four different classes (benign, DCIS, IDC, and ambiguous) with different colors on the slide. According to the model, the red region was IDC, the green region was DCIS, and the purple region was ambiguous. Purple regions usually surround the red and green regions somewhat since they represent the border of benign, IDC, or DCIS. When we focused on the regions with a high volume of purple, we discovered that these regions exhibited ambiguous histomorphological characteristics. As shown in Figure 4, MurSS identified regions with tissue artifacts as ambiguous (Figure 4a). In these areas, tumor cell nests were detected without a clear myoepithelial cell layer (Figure 4b). Additionally, the model diagnosed the lesion as vague in areas where sclerosing adenosis and small tumor cell nests were intermingled (Figure 4c).

### 4.2. Limitations and Future Work

Despite the use of MurSS, the performance regarding DCIS remains low. This low performance is likely due to the minimal amount of DCIS data available for training, accounting for only 0.5% of all pixels. This data imbalance can negatively impact the performance of deep learning models [30]. We conducted multiple experiments, such as oversampling the DCIS, weighted cross-entropy loss, etc. The results are presented in Tables S3–S5. Collecting a significant amount of DCIS data is necessary. Additionally, our model demonstrated a gap in performance between the validation and test sets. Differences in the intensity of data scanning and staining for each institution may account for the observed variations. Studies have shown that the staining technique significantly affects performance [31]. A normalization method for data collected from various institutions is needed to address this issue. The normalization method still presents a challenge for future work. MurSS and other deep learning models require significant improvement to be applicable in medical image analysis. A multi-resolution approach based on pathology data understanding, rather than solely learning with a single resolution, is a promising strategy. Also, in medical image analysis, where uncertain results or data can have significant consequences, it is advisable to acknowledge and address these uncertainties. Our proposed solution, SSM, offers one possible approach to address these challenges. It also leverages the accurate segmentation of invasive areas to automate tumor type and histologic grade analyses in future research.

## 5. Conclusions

This paper presents MurSS to address the challenge of training deep learning models with gigapixel-sized, high-resolution WSIs. To reduce computational cost, each WSI is divided into smaller patches. However, this approach may cause the model to focus solely on local information rather than the entire WSI, potentially leading to reduced performance. To address these issues, MurSS overcomes the problem of relying solely on local information by using both local content information, obtained at a high resolution, and global contextual information, obtained at a low resolution.

Additionally, for stable training and accurate diagnosis, deep learning models require the accurate labeling of morphologically similar but distinct conditions, such as DCIS and IDC. This histomorphological similarity can result in inconsistent annotations, even among expert pathologists. To address this, SSM is proposed to improve performance by rejecting inaccurate annotations caused by morphological similarities. Therefore, MurSS can identify ambiguous regions and allow pathologists to review them. The results show that MurSS achieved an improvement in mIoU ranging from 0.0717 to 0.1234 compared to existing models. MurSS also achieved a pixel-level accuracy of 98.28%, which is a 2.61% to 4.25% improvement over previous models. Based on these findings, we propose research avenues for addressing the limitations of medical imaging.

## Figures and Tables

**Table 1:** Sample confusion matrix for IDC.

Confusion Matrix for IDC		Predicted Values		
		**Benign**	**DCIS**	**IDC**
Pathologists	Benign	TN	FN	FP
	DCIS	FN	TN	FP
	IDC	FN	FN	TP

TN: true negative; FN: false negative; FP: false positive; TP: true positive; DCIS: ductal carcinoma in situ; IDC: invasive ductal carcinoma.

**Table 2:** Performance results. Comparative analysis of various segmentation models. The performance metrics include pixel-level accuracy, mean Intersection over Union (mIoU), and class-specific Intersection over Union (IoU).

Model	Coverage Ratio	Overall Measure (95% CI)		Intersection over Union (IoU)		
		Accuracy (%)	mIoU	Benign	DCIS	IDC
U-Net *	1.0	94.77 (93.39, 95.89)	0.6651 (0.6339, 0.6970)	0.9470	0.3740	0.6743
U-Net ^†^	1.0	94.97 (93.75, 95.96)	0.6497 (0.6210, 0.6765)	0.9504	0.3360	0.6626
U-Net ^‡^	1.0	94.09 (92.48, 95.35)	0.6375 (0.6040, 0.6688)	0.9393	0.3463	0.6268
HRNet *	1.0	95.58 (94.57, 96.35)	0.7005 (0.6593, 0.7312)	0.9546	**0.4361**	0.7106
DeepLabV3 *	1.0	95.60 (94.71, 96.35)	0.7013 (0.6631, 0.7341)	0.9555	0.4360	0.7123
ICNet *^†‡^	1.0	94.55 (93.49, 95.40)	0.6714 (0.6349, 0.7016)	0.9437	0.4008	0.6698
DMMN *^†‡^	1.0	94.42 (93.23, 95.36)	0.6424 (0.6170, 0.6699)	0.9436	0.3287	0.6549
MurAN *^†^	1.0	95.88 (94.85, 96.71)	0.7055 (0.6640, 0.7399)	0.9577	0.4260	0.7328
MurSS *^†^	**0.95**	**96.88** **(95.97, 97.62)**	**0.7283** **(0.6865, 0.7640)**	**0.9690**	0.4324	**0.7833**
MurSS *^†^	0.90	97.09 (96.13, 97.85)	0.7356 (0.6970, 0.7705)	0.9707	0.4363	0.7999
*MurSS* *^†^	*0.80*	*98.30* *(97.53, 98.86)*	*0.7603* *(0.7067, 0.8061)*	*0.9839*	*0.4485*	*0.8487*

*: high-resolution input (5.0×); ^‡^: mid-resolution input (2.5×); ^†^: low-resolution input (1.25×). **Bold**: The model utilized throughout the entirety of the manuscript. *Italic*: The state-of-the-art performance.

## Data Availability

The code used in this article cannot be published due to privacy but can be obtained from the corresponding author upon reasonable request.

## Supplementary Materials

The following supporting information can be downloaded at https://www.mdpi.com/article/10.3390/bioengineering11050463/s1, Table S1: Validation Result from the Best Epoch; Table S2: Test Result Statistical Analysis; Table S3: Sampled Test about Oversampling DCIS; Table S4: Sampled Test about Weighted Cross Entropy Loss; Table S5: Sampled Test about Data Uncertainty; Figure S1a–c: VIsualization Results.

## Abbreviations

The following abbreviations are used in this manuscript:

Accuracy	pixel-level accuracy (%)
AdaIN	adaptive instance normalization
BRCA	breast invasive carcinoma
CI	confidence interval
CNN	convolutional neural network
DCIS	ductal carcinoma in situ
H&E	hematoxylin and eosin
IDC	invasive ductal carcinoma
IoU	Intersection over Union
KUMC-Guro	Korea University Medical Center, Guro Hospital
mIoU	mean Intersection over Union
MurAN	multi-resolution adaptive normalization
MurSS	multi-resolution selective segmentation
SSM	selective segmentation method
TCGA	The Cancer Genome Atlas
WSI	whole-slide image
