# Supplementary material for: MurSS: A Multi-Resolution Selective Segmentation Model for Breast Cancer
Source: Bioengineering (Basel). 2024 May 7;11(5):463. doi: 10.3390/bioengineering11050463 (PMC11117971; doi:10.3390/bioengineering11050463)
Supplement: Supplementary file 1 [file bioengineering-11-00463-s001.zip › bioengineering-2964424-supplementary.pdf]

Supplementary

MurSS: Multi-resolution Selective Segmentation Model for Breast Cancer

Table S1. Validation Result from the Best Epoch. This section presents the validation performance of multiple deep-learning models. Validation exhibits a comparable trend to Test, with the highest performance achieved on MurSS with a coverage ratio of 0.8. MurAN also performs well by overall measure compared to other deep learning models.

Model	Magnification (Coverage ratio)	Overall Measure		Intersection over Union (IoU)		
		Accuracy (%)	mIoU	Benign	DCIS	IDC
UNet	50x	92.90	0.7426	0.9118	0.4944	0.8216
UNet	25x	93.56	0.7669	0.9165	0.5473	0.8370
UNet	12.5x	90.44	0.6906	0.8721	0.4404	0.7593
HRNet	50x	94.88	0.7431	0.9272	0.4332	0.8689
DeepLabV3	50x	94.35	0.7209	0.9223	0.3848	0.8556
ICNet	50x, 25x, 12.5x	93.43	0.7864	0.9118	0.6050	0.8425
DMMN	50x, 25x, 12.5x	93.56	0.7693	0.9166	0.5490	0.8424
MurAN	50x, 12.5x	96.33	0.8151	0.9455	0.5950	0.9049
Murss	50x, 12.5x (0.95)	98.04	0.8499	0.9717	0.6298	0.9483
MurSS	50x, 12.5x (0.90)	98.23	0.8579	0.9742	0.6459	0.9537
**MurSS**	**50x, 12.5x (0.80)**	**99.36**	**0.9114**	**0.9911**	**0.7616**	**0.9815**

Table S2. Test Result Statistical Analysis. 95% Confidence Intervals (CIs) are calculated using 95 reconstructions and 1000 bootstraps about the performance difference (Model 1 - Model 2) per slide to evaluate the superiority between the models. When comparing MurAN to other models, we found that it did not show statistically significant performance superiority compared to DeepLabV3 and HRNet models. However, it did show a statistically significant difference compared to U-Net, ICNet, and DMMN models. For MurSS, we observed statistically significant performance gains in pixel-level accuracy and mIoU compared to the other deep learning models.

Model 1	Model 2	95% CI Accuracy difference (lower, upper (%p))	95% CI mIoU difference (lower, upper)
MurAN	UNet	0.48, 1.46	0.0065, 0.0353
	DeepLabV3	-0.17, 0.54	-0.0173, 0.0133
	HRNet	-0.05, 0.56	-0.0129, 0.0174
	ICNet	1.05, 1.63	0.0229, 0.0487
	DMMN	1.03, 1.82	0.0242, 0.0546
MurSS (0.95)	UNet	1.44, 2.57	0.0292, 0.0646
	DeepLabV3	0.93, 1.63	0.0120, 0.0459
	HRNet	0.82, 1.66	0.0057, 0.0436
	ICNet	2.03, 2.74	0.0478, 0.0772
	DMMN	2.03, 2.91	0.0488, 0.0840

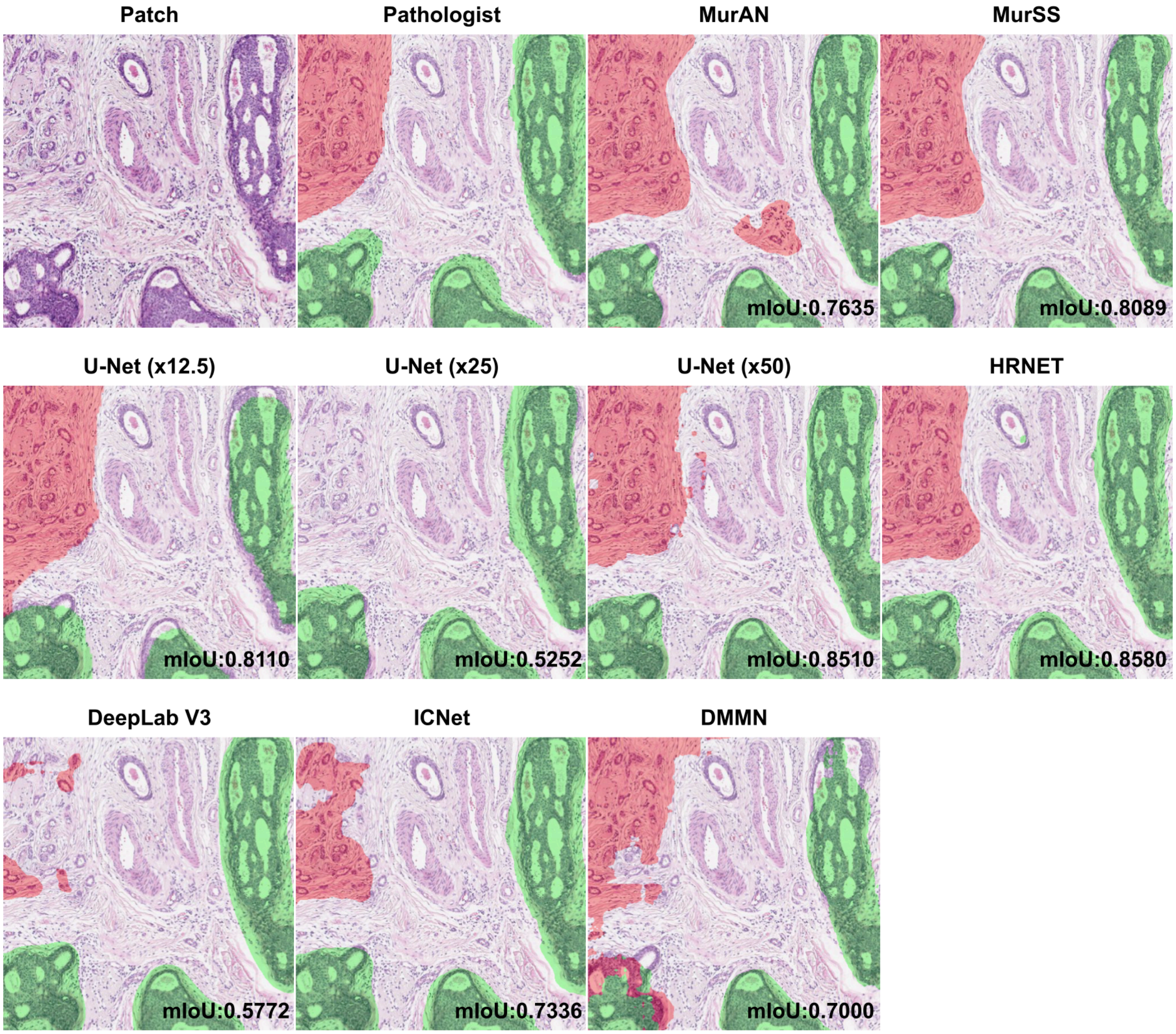

Figure S1 (a) Visualization Results. Pathologists and all deep learning models were compared. The mIoU was measured at the patch level. Green areas represented DCIS, and red areas represented IDC.

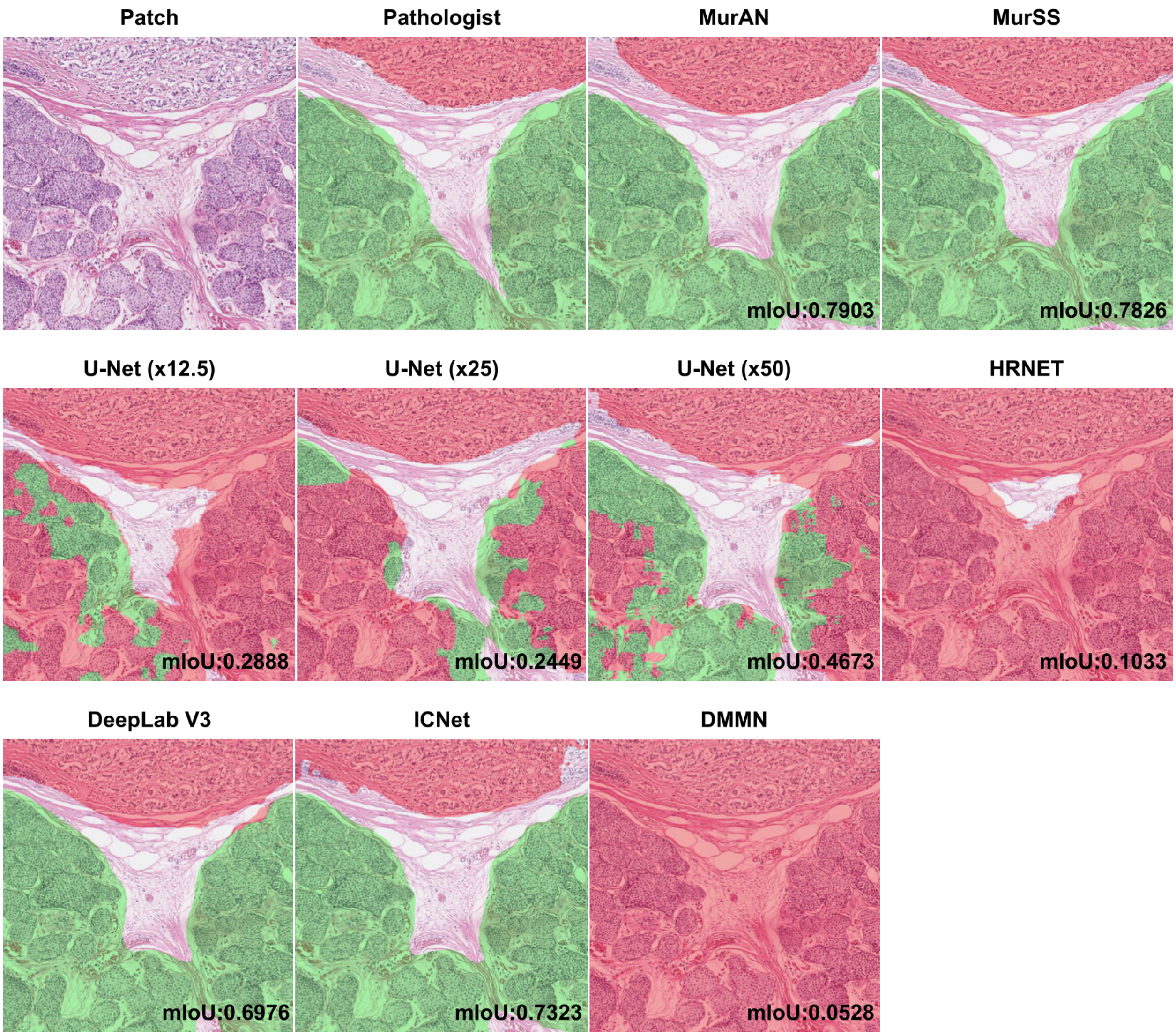

Figure S1 (b) Visualization Results. Pathologists and all deep learning models were compared. The mIoU was measured at the patch level. Green areas represented DCIS, and red areas represented IDC.

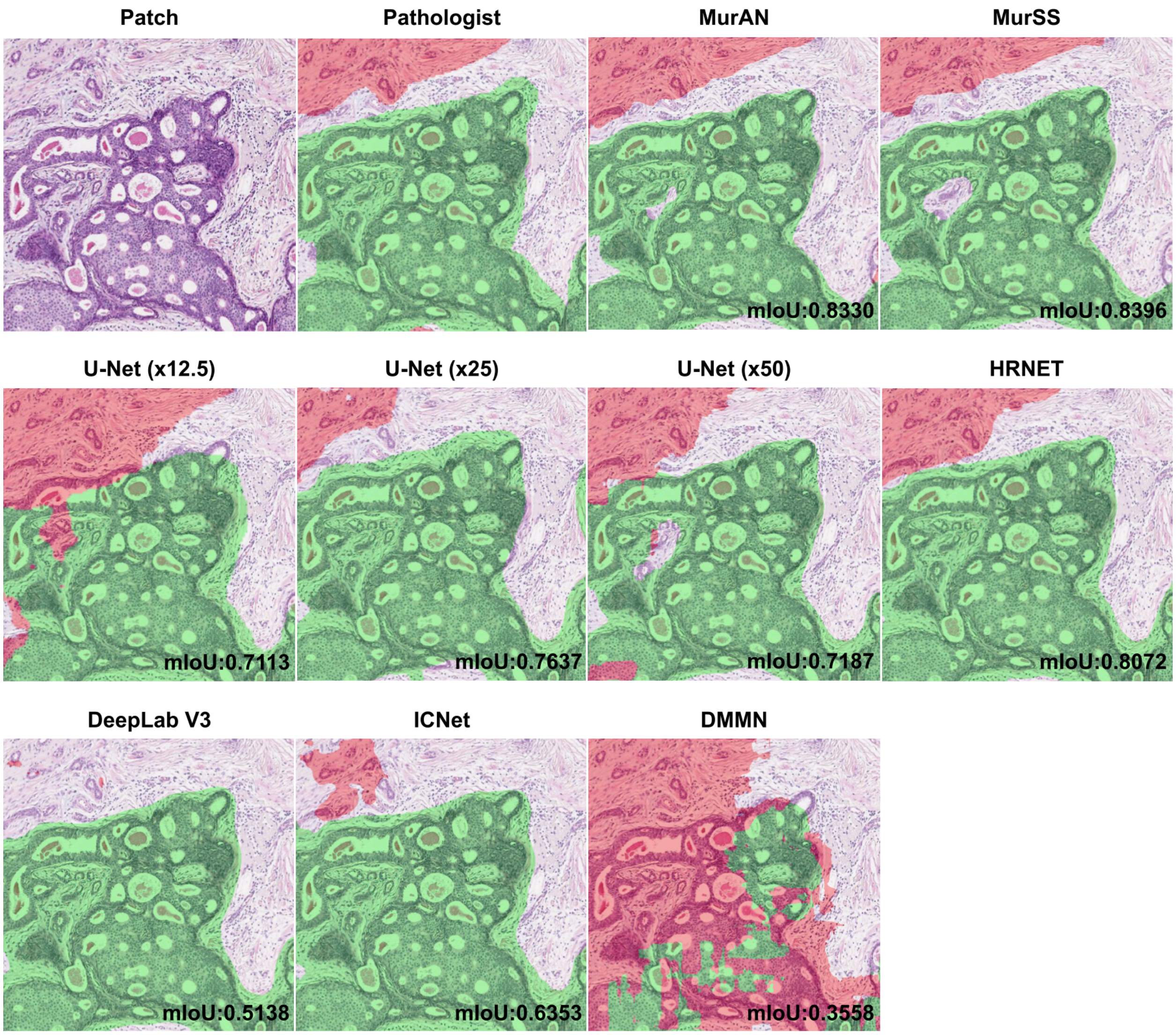

Figure S1 (c) Visualization Results. Pathologists and all deep learning models were compared. The mIoU was measured at the patch level. Green areas represented DCIS, and red areas represented IDC.

Table S3. Sampled Test about Oversampling DCIS. One hundred one slides from the TCGA dataset were selected where the annotations made by the teachers were similar. Fifty-one slides were for training, and the remaining fifty were divided equally for validation and testing. Multiple oversampling for DCIS patches due to their significantly low number of data. The same patches are utilized multiple times based on the oversample ratio. Subsequently, hard augmentations were implemented, including flipping, rotating, blurring, cropping, color jittering, and so forth. Significant performance gains were observed, up to seven-teen oversampling for DCIS. However, no further significant performance gains were observed beyond this point.

MurAN Oversampling DCIS	Overall Measure		Intersection over Union (IoU)		
	ACC (%)	mIoU	Benign	DCIS	IDC
1 times	96.2	0.765	0.945	0.438	0.902
9 times	96.2	0.773	0.945	0.471	0.903
17 times	96.2	0.775	0.945	0.479	0.902
25 times	96.2	0.772	0.945	0.475	0.903
33 times	96.2	0.774	0.946	0.473	0.903
33 times	96.2	0.774	0.946	0.472	0.902
41 times	96.2	0.773	0.945	0.471	0.903

Table S4. Sampled Test about Weighted Cross Entropy Loss. One hundred one slides from the TCGA dataset were selected where the annotations made by the teachers were similar. Fifty-one slides were for training, and the remaining fifty were divided equally for validation and testing. We attempted to balance the data by using weighted cross-entropy loss with nine oversampling for DCIS. However, as shown in the table below, this did not significantly improve performance.

MurAN Weight ratio	Overall Measure		Intersection over Union (IoU)		
	ACC (%)	mIoU	Benign	DCIS	IDC
1:1:1	96.2	0.773	0.945	0.471	0.903
1:3:3	95.7	0.766	0.938	0.465	0.894
1:9:9	94.7	0.747	0.922	0.444	0.873
1:13:13	94.3	0.738	0.915	0.434	0.915
1:3:1	96.1	0.773	0.944	0.476	0.900
1:9:1	96.1	0.765	0.945	0.448	0.902

Table S5. Sampled Test about Data Uncertainty. One hundred one slides from the TCGA dataset were selected where the annotations made by the teachers were similar. Fifty-one slides were for training, and the remaining fifty were divided equally for validation and testing. The experiment was conducted at nine times the oversampling rate compared to DCIS. MurAN + data involved removing regions with different annotation labels between pathologists and poor slide-quality patches during the training and evaluation of MurAN. We also added DCIS data by reducing the stride when extracting patches from WSIs.

Model	Overall Measure		Intersection over Union (IoU)		
	ACC (%)	mIoU	Benign	DCIS	IDC
MurAN	96.2	0.773	0.945	0.471	0.903
MurAN + data	96.0	0.789	0.942	0.527	0.899
